# Insomnia Status of Middle School Students in Indonesia and Its Association with Playing Games before Sleep: Gender Difference

**DOI:** 10.3390/ijerph18020691

**Published:** 2021-01-15

**Authors:** Septian Arief Gandaputra, Imam Waluyo, Ferry Efendi, Jiun-Yi Wang

**Affiliations:** 1 Physiotherapy Program, Universitas Binawan, Jakarta 13630, Indonesia; septian@binawan.ac.id (S.A.G.); scientist.com_antoniwa@mail.com (I.W.); 2 Department of Healthcare Administration, College of Medical and Health Science, Asia University, Taichung 413, Taiwan; 3 Faculty of Nursing, Universitas Airlangga, Surabaya 60115, Indonesia; ferry-e@fkp.unair.ac.id; 4 Department of Medical Research, China Medical University Hospital, China Medical University, Taichung 404, Taiwan

**Keywords:** online games, playing games before sleep, insomnia, adolescents, middle school

## Abstract

The excessive use of online gaming before sleep in adolescents could be a risk factor of sleep disorders such as insomnia. This study aimed to assess the association between playing online games before sleep and insomnia based on gender perspective among middle school students in Indonesia. This was a retrospective study and the data came from a cross-sectional survey that included 315 of Grade 8 and 9 students from four middle schools in Jakarta, Indonesia. Self-reported data were collected via a structured questionnaire, which consisted of demographic variables, sleep duration per day, frequency of playing online games before sleep and the Insomnia Severity Index (ISI). Insomnia status was classified into “none or mild” and “moderate to severe” according to ISI score. The proportions of male and female students who often or always played online games before sleep were 50.3% and 21.7%, respectively. Grade 9 students were more likely to have moderate to severe insomnia than Grade 8 students for males (odds ratio—OR = 4.34, *p* = 0.005) but not for females (OR = 0.69, *p* = 0.363). However, female students who often or always played online games before sleep were more likely to have moderate to severe insomnia (OR = 4.60, *p* = 0.001); such an association was relatively smaller for male students (OR = 3.09, *p* = 0.061). Gender differences should be taken into account when developing health education or interventions.

## 1. Introduction

Online gaming has become a very popular industry in this decade, especially in South East Asia [1]. In Indonesia, revenue in the mobile games segment is projected to reach US $712M in 2020 [2]. In addition, according to the results reported by APJII (Indonesian Internet Service Providers Association), the prevalence of Internet use has increased from 34.9% (88.1 million users) in 2014 to 51.8% (132.7 million users) in 2016 and 73.7% (193 million users) in 2019. Meanwhile, the current prevalence in 2019 of online game users is 16.5% [3]. Some studies have reported that online gaming could have positive effects for adolescents, such as exhibiting superior visual, spatial and attention skills [4,5] and strong social ties [6]. It could be related only when gamers engage in online activities that continue beyond the game and extend these with offline activities [7]. However, the negative impacts have been a great concern. Excessive online gaming is suggested to be categorized as a concept of nonsubstance addiction or impulse-controlled disorder (ICD) [8] because there is repeated behavioral engagement, and it has been indicated to increase negative consequence. In addition, excessive use of Internet games is thought to be related to impulse control, reward processing and somatic representations caused by abnormal resting in the orbito-frontal cortex, striatum and sensory areas [9].

Many studies also reported that excessive of online gaming is associated with sleep quality in adolescents [10,11,12,13,14,15,16]. Playing online games at bedtime/before sleep in adolescents, which is prolonged, would predict to lead to the insomnia condition [11]. Furthermore, long exposure of insomnia condition in adolescents is associated with several problems such as being an obstacle of brain maturity stages [17,18,19], mental problems [20,21], social isolation [22], academic performance and reduced performance in next day’s activities.

Related to gender differences, studies have been conducted regarding sleep disorders and their relationship to Internet gaming in adolescents, both male and female [10,13,23,24,25]. Most studies pointed out that males are more likely to use their devices for gaming and have less sleep. Meanwhile, multimedia applications and social interaction services engaged females. Nowadays, however, game developers around the world build the game content to be attracted and less restricted to a specific gender. One study [8] examined gender differences in playing online games among public school students. The results reveal that female students also tended to play games similarly to male students. It also reported that female students were more frequently to report playing games fewer than 7 h per week, while 14% of male students reported playing ≥20 h per week.

In Indonesia, a large proportion population survey reported that, among 3411 respondents aged 16 and over, 34% of Indonesian female respondents played casual games (video game without investing significant time, spontaneously, irregularly or infrequently). In comparison, role-playing games and multiplayer games were most popular among male gamers, with 40% of respondents [2].

Previous studies have described relationships of game playing behaviors and age. For instance, a study compared the duration of playing games between adolescents and adults. In general, the results of the study found that adolescents (under the age of 20) played games approximately 26.3 h a week or 4.36 h per day which was significantly higher than adults [26]. For late adolescents and its relation to the gaming addiction, some studies identified that young players (range 12–18 years) and those with student identify had a tendency to play more hours of intensive online games per week [27,28].

Furthermore, some negative outcomes due to uncontrolled habits on technology among adolescents has been recognized in the literature. While males are recognized as more likely to have gaming addiction [12,16,24,29], the number of female gamers continues growing [2,8,30]. Research on the role and behavior differences between male and female adolescents in the excessive game playing and its impact on health outcomes such as insomnia is essential. Therefore, in the present study, we aimed to assess the gender differences in insomnia, online gaming behavior before sleep and their associations among middle school students in Indonesia.

## 2. Materials and Methods

### 2.1. Participants and Sampling Procedures

This was a retrospective study and the data came from a cross-sectional survey. A three-stage non-random selection was carried out in determining a sample of middle school students in the East Jakarta district (the capital city of Indonesia). Four middle schools (1 private and 3 public) in the urban area were enrolled by considering their accessibility. Afterward, the criteria for the subject of school students who meet the requirements in this study were determined. The inclusion criteria specified were participants having a device such as a gaming computer or smartphone with a minimum of android operation or something compatible with online game applications and students in Grade 8 or 9 with an age range of 13–15 years old. Participants who refused to join in the study were excluded. The remaining participants were then entered into a list in order and randomly selected by systematic sampling. In total, 315 students, 149 males and 166 females, were enrolled in the current study. Informed consents from participants and ethical approval for this study from the Binawan University Human Research Ethics Committee (No. 020/EP/KE/UBINAWAN-BIN/VI/2018) were obtained.

### 2.2. The Exposure Variable and Outcome Variable

A structured questionnaire was distributed to the enrolled participants. All responses were self-reported. The exposure or major independent variable in the present study was the playing games behavior before sleep. In this study, we focused on smartphone use in playing online games because, as a medium, smartphones are more affordable and accessible. Although offline games are another type of games, it was not measured. From the questionnaire, the question: “How often do you use smartphone to play online games for 1 h or more before sleep?” was used to measure a participant’s online gaming behavior before sleep. One hour or more of playing online games suggests an excessive light exposure before sleep and was adopted in previous studies [31,32]. There were five options: never, rarely, sometimes, often and always. To avoid too many categories in a variable, similar to a previous study [33], we re-classified them into three levels for all the analyses: never or rarely, sometimes and always or often.

The outcome or response variable was the insomnia status which was measured by using the Insomnia Severity Index (ISI). Good validity and reliability of this instrument were confirmed by previous study [34]. The ISI can be used to evaluate onset severity, sleep maintenance and problems after waking up, sleep dissatisfaction, sleep difficulties with daytime dysfunction, other people’s opinions of sleep problems experienced and suffering due to sleep difficulties. There were seven questions with a five-point scoring system (scores 0–4 indicate from no problem to a serious problem). The total score obtained ranged 0–28. The score can be qualified as: 0–7 clinically insomnia was not significant, 8–14 insomnia threshold, 15–21 clinical insomnia (moderate severity) and 22–28 clinical insomnia (severe). In the present study, it was reclassified into two levels (none or mild vs. moderate to severe) for binary logistic regression analysis.

### 2.3. Other Independent Variables

Several other independent variables included in the study were the school grade (Grades 8 and 9), gender (male and female), age (years), sleep duration and smartphone use. For sleep duration, participants were asked to respond the question “How many hours do you usually sleep over night”. The responses were: (1) less than 8 h; and (2) 8 h or more. They were adopted from the National Sleep Foundation guideline about the sleep time for adolescents [35]. With regard to smartphone use, we classified it similar to in previous studies [10]. Participants were asked to respond the question “How many hours do you use smartphone per day”. Reponses were 2 h or less, 3 h or more but less than 5 h and 5 h or more. They were reclassified into two levels: (1) ≤2 h; and (2) ≥3 h.

### 2.4. Statistical Analysis

All data analyses were performed by using IBM SPSS Statistics v.22(IBM Corp, Armonk, NY, USA). Descriptive statistics such as number and percentage were used for categorical data; mean and standard deviation were calculated for continuous data. Chi-square test was performed to compare levels of the studied variables between students with no or mild insomnia and moderate to severe insomnia, stratified by gender. The logistic regression analysis was conducted to assess the associations between variables and insomnia status among male and female students. The significance level was set at 0.05 for all tests.

## 3. Results

In total, 315 participants (149 males, 166 females) aged between 12 and 15 years were recruited from four middle schools. The descriptive statistics are shown in Table 1. The results show that there was no significant difference in sleep duration: 107 males (71.8%) and 118 females (71.1%) usually slept less than 8 h. Compared to female students, male students reported a higher proportion of always or often playing online games before sleep (50.3% vs. 21.7%, *p* < 0.001), but they reported a lower proportion of smartphone use (54.4% vs. 75.9%, *p* < 0.001). Moreover, proportions of male students having moderate and severe insomnia were 14.8% and 2.0%, respectively, while they were 17.5% and 2.4%, respectively, for female students. There was no significant difference in the insomnia status between male and female students.

Table 2 explains the bivariate associations between variables (school grade, smartphone use, sleep duration and playing games before sleep) and the insomnia status among the male and female students. For male students, it shows that 24.0% of Grade 9 students have moderate to severe insomnia, which was significantly higher than in Grade 8 students (χ^2^ = 5.64, *p* = 0.027). Male students who always or often played games before sleep had higher but non-significant likelihood to have moderate to severe insomnia (22.7%), compared to those who sometimes (7.7%) and never or rarely (14.3%) played games before sleep. For female students, on the other hand, those who always or often played games before sleep had significantly higher likelihood to have moderate to severe insomnia (41.7%), compared to those who sometimes (13.9%) and never or rarely (13.8%) played games before sleep (χ^2^ = 13.70, *p* = 0.001).

Table 3 summarizes results from the logistic regression analyses for male and female students, respectively. They are similar to those from bivariate analyses. Male students in Grade 9 were 4.34 times as likely as male Grade 8 students to have moderate to severe insomnia (OR = 4.34, *p* = 0.005). Male students who always or often played games before sleep tended to be more likely to have moderate to severe insomnia (OR = 3.09, *p* = 0.061), compared to those who never played games before sleep. For female students, those who always or often played games before sleep were 4.60 times as likely as those who never played games before sleep to have moderate to severe insomnia (OR = 3.09, *p* = 0.061).

## 4. Discussion

The present study collected data from four middle schools in East Jakarta, Indonesia and found that female students had more smartphone use per day than male students. However, male students played online games before sleep more often. The results reveal that female students who always or often played games before sleep were more likely to have moderate to severe insomnia. Although a similar result was observed for male students, the OR was a little smaller and did not achieve statistical significance.

Our results suggest that the frequency of playing online games before sleep is associated with insomnia in adolescents. The behavior of playing games before sleep has been reported to be a reason for shortened duration and delayed timing of sleep [36]; it could be prolonged and thus would be associated with sleep disturbance such as insomnia [10,11,37]. Insomnia includes some conditions such as long sleep latency, frequent waking at night or prolonged waking periods during sleep [18].

The underlying mechanism of the association between playing online games before sleep and insomnia could be explained by previous studies. For instance, the use of smartphones or screen time before bedtime may result in light exposure that could affect circadian rhythm (internal process that regulates the sleep–wake cycle) and alertness [36,37]. Other studies observed the light exposure of screen time before sleep would provoke the melatonin suppression controlled by hypothalamus and the process occurs after 2 h of use [32,38,39]. Alternatively, one study observed that the effect to both psychological and physiological arousal (measured by systolic blood pressure, diastolic blood pressure and heart rate) due to the content of games (violent vs. non-violent) may also interfere with the ability to fall and stay asleep. The study involving 395 participants showed that exposure to violent video games increased physiological arousal [40]. To link all of those mechanism, one study proved that prolonged gaming before sleep produced decreases in sleep efficiency, which was specified by 17 ± 8 min increases of sleep-onset latency among the subjects [11]

The study also found that male students always or often played online games for more than 1 h before sleep and the proportion was over twice as likely as female students (50.3% vs. 21.7%). This result is in line with a previous study which observed that males tend to play games for 2–3 h after finishing their homework [41]. To compare the tendency of gaming use before sleep between males and females, several studies in neurobiological development might offer more specific context about adolescent brain development and behavioral addiction. For instance, behavioral addiction model, also known as dual-systems model (high sensation seeking tendencies and low impulse control) in development, has been observed to be related to the pattern of risky behavior [42,43], where males had higher levels of sensation seeking and lower levels of impulse control than females [44]. More specifically, excessive use of Internet games is thought to be related to impulse control, reward processing and somatic representations caused by abnormal resting in the orbito-frontal cortex, striatum and sensory areas [9]. This model has also been used to evaluate the association between excessive use in Internet gaming and delayed sleep time [8,45,46].

It is worth noting that game developers have created many gaming genres, making them more attractive this decade, and online or video games tend to be attractive to both males and females. This study revealed that almost half of female students sometimes or more frequently played online games before sleep, which is similar to a previous study [8]. The argument might also be supported by a statistics survey [2]. The survey described the current online game genres such as role-playing games (RPG/MORPG/MMORPG), sports, real-time strategy, first person shooter, battle royal games, action/adventure, casino and card games, casual games, fighting games, simulation games, racing games and many others. Those genres might also tend to impress females to play online games.

This study revealed that there was an association for female students between playing online games before sleep and insomnia status (OR = 4.60). The strength of such an association for male students, however, was a little smaller (OR = 3.09) and did not reach statistical significance. This is an important finding, suggesting that female students who frequently played online games before sleep were more likely to having insomnia experience. In contrast, several other studies examining gender differences in playing online games before sleep and their association with sleep disorders found that males use smartphones more often to play online gaming than females [8,24,25]. While some studies showed that female gamers had shorter duration of online gaming [41,47], studies also revealed that females were 1.28 times as likely as males to have sleep problem [25,48]. The physiological links between puberty and hormonal changes may partially explain the additional factors among female gamers on insomnia condition. Females’ reproductive hormones such as progesterone and estrogen change in sleep during the menstrual cycle [48]. Many females have sleep complaints during the menstrual cycle (follicular and luteal) because the increasing of estrogen hormone during this phase (luteal phase) might cause the turnover of the neurotransmitter norepinephrine and decrease rapid eye movement (REM) latency [49], thus it becomes a sleep disorder. Moreover, it has been shown that females were more likely to have severe somatic, pain and anxiety symptoms compared to males [8]. Thus, they could be more vulnerable to depression, anxiety and psychological distress [50], which would affect sleep disturbances or induce insomnia symptoms such as daytime fatigue and reduced daytime performance [51,52].

This study found that the proportions of students using devices (smartphones) for 3 h or more were 54.4% and 75.9% in males and females, respectively. It implies that the total duration of using smartphones was significantly higher in female students. Many studies have suggested that playing devices for hours is a concern related to poor sleep and physical (including vitality) and mental health [10,53,54]. People spending time using smartphones can have multiple purposes, such as entertainment, social connection, time-killing, checking information and so on [55].

Some studies observed that males are more likely to use smartphones for engaging with harmful content such as pornography and gambling [53,56,57] or online gaming [58], whereas girls use smartphones more often to communicate with others, either through social network sites or directly chatting or texting. They also explore multimedia applications and social interaction services frequently [53,58]. Problem-solving skills may explain the gender difference in smartphone use. For instance, males tend to use avoidance coping mechanisms to tolerate frustration and seek sensation, whereas females seek help from others to tolerate emotional discomfort [59,60].

These gender differences reveal that more studies are required to understand and discuss underlying difference between male and female students. It would also be valuable to study whether other variables such as mood, anxiety disorders and problematic mobile phone use could affect the male and female groups differently.

### Limitations

There are some limitations in the present study. First, participants in the sample were enrolled from four middle schools; however, they were not enrolled at random. The four schools are located in East Jakarta, which is an urban area with dense population. Comparing to the whole population in Indonesia, the present sample might come from families with higher socioeconomic status. It implies that the results should be inferred with caution. Second, the study did not measure offline gaming behavior but had added duration of smartphone use in the analytical models for adjustment, which is expected to reduce the level of potential bias. Moreover, the study focused on the frequency of playing online games before sleep; however, a variety of activity before sleep might also be associated with insomnia, such as the use of social media, creating video content, listening to music, watching videos or movies, etc. Without measuring all possible activities, the results might be biased.

## 5. Conclusions

The findings based on the study participants from four middle schools in Indonesia suggest that gaming behaviors before sleep are more common in male students. Playing online games before sleep is a risk factor of insomnia. However, the extent of association between insomnia and gaming behaviors before sleep of male students is just comparable to or slightly smaller than female students. Educational personnel and parents may want to put more efforts on preventing excessive online gaming among adolescents. In addition, gender differences should be considered when developing health education or interventions.

## Figures and Tables

**Table 1:** Characteristic and variable distributions of the study participants, stratified by gender.

Variable ^a^	Total Sample (*n* = 315)	Males (*n* = 149, 47.3%)	Females (*n* = 166, 52.7%)	*p*-Value
Age (years)				
Mean ± SD	13.59 ± 0.77	13.63 ± 0.817	13.56 ± 0.73	0.795
Min-max	12–15	12–15	12–15	
Grade				0.417
8	154 (48.9)	74 (49.7)	80 (48.2)	
9	161 (51.1)	75 (50.3)	86 (51.8)	
Smartphone use (hours/day)				<0.001
≤2	108 (34.3)	68 (45.6)	40 (24.1)	
≥3	207 (65.7)	81 (54.4)	126 (75.9)	
Sleep duration (hours/day)				0.89
<8	225 (71.4)	107 (71.8)	118 (71.1)	
≥8	90 (28.6)	42 (28.2)	48 (28.9)	
Online gaming before sleep (≥1 h)				<0.001
Never or rarely	129 (41.0)	35 (23.5)	94 (56.6)	
Sometimes	75 (23.8)	39 (26.2)	36 (21.7)	
Always or often	111 (35.2)	75 (50.3)	36 (21.7)	
Insomnia status (ISI score)				0.48
No clinical insomnia (0–7)	124 (39.4)	65 (43.6)	59 (35.5)	
Subthreshold (8–14)	133 (42.2)	59 (39.6)	74 (44.6)	
moderate (15–21)	51 (16.2)	22 (14.8)	29 (17.5)	
Severe (22–28)	7 (2.2)	3 (2.0)	4 (2.4)	

SD—standard deviation; ISI—insomnia severity index. ^a^ Data are presented as *n* (%).

**Table 2:** The bivariate associations between insomnia status and independent variables, stratified by gender.

Variable ^a^	Insomnia Status in Males				Insomnia Status in Females			
	None or Mild	Moderate to Severe	χ^2^	*p*-Value	None or Mild	Moderate to Severe	χ^2^	*p*-Value
Grade			5.64	0.027			0.67	0.442
8	67 (90.5)	7 (9.5)			62 (77.5)	18 (22.5)		
9	57 (76.0)	18 (24.0)			71 (82.6)	15 (17.4)		
Smartphone use (hours/day)			0.49	0.516			0.00	1.000
≤2	55 (80.9)	13 (19.1)			32 (80.0)	8 (20.0)		
≥3	69 (85.2)	12 (14.8)			101 (80.2)	25 (19.8)		
Sleep duration (hours/day)			0.26	0.808			1.11	0.292
<8	88 (82.2)	19 (17.8)			97 (82.2)	21 (17.8)		
≥8	36 (85.7)	6 (14.3)			36 (75.0)	12 (25.0)		
Playing games before sleep (≥1 h)			4.32	0.115			13.7	0.001
Never or rarely	30 (85.7)	5 (14.3)			81 (86.2)	13 (13.8)		
Sometimes	36 (92.3)	3 (7.7)			31 (86.1)	5 (13.9)		
Always or often	58 (77.3)	17 (22.7)			21 (58.3)	15 (41.7)		

^a^ Data are presented as *n* (%).

**Table 3:** Logistic regression analysis for exploring associations between insomnia status and independent variables, stratified by gender.

Variable	Males		*p*-Value	Females		*p*-Value
	OR	95% CI		OR	95% CI	
Grade						
8	1.00			1.00		
9	4.34	(1.55, 12.15)	0.005 **	0.69	(0.30, 1.55)	0.363
Smartphone use (hours/day)						
≤2	1.00			1.00		
≥3	0.52	(0.20, 1.33)	0.170	1.1	(0.43, 2.81)	0.848
Sleep duration (hours/day)						
<8	1.00			1.00		
≥8	0.74	(0.25, 2.20)	0.592	1.33	(0.57, 3.14)	0.511
Playing games before sleep						
Never or rarely	1.00			1.00		
Sometimes	0.68	(0.14, 3.28)	0.635	1.07	(0.35, 3.28)	0.909
Always or often	3.09	(0.95, 10.07)	0.061	4.6	(1.87, 11.28)	0.001 **

OR, odds ratio; 95% CI, 95% confidence interval. ** *p* < 0.01.

## Data Availability

The student survey data presented in this study are not publicly available due the data use agreements.

## References

[1] Post T.A. (2020). The Gaming Explosion in Southeast Asia. https://theaseanpost.com/article/gaming-explosion-southeast-asia.

[2] (2020). Statista, Mobile Games: Core Country Data Based In-Depth Analysis. Indonesia. https://www.statista.com/outlook/211/120/mobile-games/indonesia.

[3] APJII (2016). Indonesian Internet User Profiles (Profil Pengguna Internet Indonesia). https://apjii.or.id/downfile/file/surveipenetrasiinternet2016.pdf.

[4] Feng J., Spence I., Pratt J. (2007). Playing an action video game reduces gender differences in spatial cognition. Psychol. Sci..

[5] Subrahmanyam K., Greenfield P.M. (1994). Effect of video game practice on spatial skills in girls and boys. J. Appl. Dev. Psychol..

[6] Phillips C.A., Rolls S., Rouse A., Griffiths M.D. (1995). Home video game playing in schoolchildren: A study of incidence and patterns of play. J. Adolesc..

[7] Trepte S., Reinecke L., Juechems K. (2012). The social side of gaming: How playing online computer games creates online and offline social support. Comput. Hum. Behav..

[8] Desai R.A., Krishnan-Sarin S., Cavallo D., Potenza M.N. (2010). Video-gaming among high school students: Health correlates, gender differences, and problematic gaming. Pediatrics.

[9] Park H.S., Kim S.H., Bang S.A., Yoon E.J., Cho S.S., Kim S.E. (2010). Altered regional cerebral glucose metabolism in internet game overusers: A 18 F-fluorodeoxyglucose positron emission tomography study. CNS Spectr..

[10] Hysing M., Pallesen S., Stormark K.M., Lundervold A.J., Sivertsen B. (2013). Sleep patterns and insomnia among adolescents: A population-based study. J. Sleep Res..

[11] King D.L., Gradisar M., Drummond A., Lovato N., Wessel J., Micic G., Douglas P., Delfabbro P. (2013). The impact of prolonged violent video-gaming on adolescent sleep: An experimental study. J. Sleep Res..

[12] Lam L.T. (2014). Internet gaming addiction, problematic use of the internet, and sleep problems: A systematic review. Curr. Psychiatry Rep..

[13] Lin P.-H., Lee Y.-C., Chen K.-L., Hsieh P.-L., Yang S.-Y. (2019). The Relationships between sleep quality and internet addiction among female college students. Front. Neurosci..

[14] Munezawa T., Kaneita Y., Osaki Y., Kanda H., Minowa M., Suzuki K., Higuchi S., Mori J., Yamamoto R., Ohida T. (2011). The association between use of mobile phones after lights out and sleep disturbances among Japanese adolescents: A nationwide cross-sectional survey. Sleep.

[15] Rehbein F., Psych G., Kleimann M., Mediasci G., Mößle T. (2010). Prevalence and risk factors of video game dependency in adolescence: Results of a German nationwide survey. Cyberpsychol. Behav. Soc. Netw..

[16] Mei X., Zhou Q., Li X., Jing P., Wang X., Hu Z. (2018). Sleep problems in excessive technology use among adolescent: A systemic review and meta-analysis. Sleep Sci. Pract..

[17] O’Brien L.M., Gozal D. (2004). Neurocognitive dysfunction and sleep in children: From human to rodent. Pediatric Clin..

[18] Roth T. (2007). Insomnia: Definition, prevalence, etiology, and consequences. J. Clin. Sleep Med..

[19] Spiegel K., Tasali E., Penev P., Cauter E.V. (2004). Brief communication: Sleep curtailment in healthy young men is associated with decreased leptin levels, elevated ghrelin levels, and increased hunger and appetite. Ann. Intern. Med..

[20] Kuss D.J., Griffiths M.D., Binder J.F. (2013). Internet addiction in students: Prevalence and risk factors. Comput. Hum. Behav..

[21] Roberts R.E., Duong H.T. (2014). The prospective association between sleep deprivation and depression among adolescents. Sleep.

[22] Colwell J., Kato M. (2003). Investigation of the relationship between social isolation, self-esteem, aggression and computer game play in Japanese adolescents. Asian J. Soc. Psychol..

[23] Chen B., Liu F., Ding S., Ying X., Wang L., Wen Y. (2017). Gender differences in factors associated with smartphone addiction: A cross-sectional study among medical college students. BMC Psychiatry.

[24] Lange K., Cohrs S., Skarupke C., Görke M., Szagun B., Schlack R. (2017). Electronic media use and insomnia complaints in German adolescents: Gender differences in use patterns and sleep problems. J. Neural Transm..

[25] Yang S.-Y., Lin C.-Y., Huang Y.-C., Chang J.-H. (2018). Gender differences in the association of smartphone use with the vitality and mental health of adolescent students. J. Am. Coll. Health.

[26] Griffiths M.D., Davies M.N., Chappell D. (2004). Online computer gaming: A comparison of adolescent and adult gamers. J. Adolesc..

[27] Smahel D., Blinka L., Ledabyl O. (2008). Playing MMORPGs: Connections between addiction and identifying with a character. Cyberpsychol. Behav..

[28] Hussain Z., Griffiths M.D., Baguley T. (2012). Online gaming addiction: Classification, prediction and associated risk factors. Addict. Res. Theory.

[29] Greenberg B.S., Sherry J., Lachlan K., Lucas K., Holmstrom A. (2010). Orientations to video games among gender and age groups. Simul. Gaming.

[30] Entertainment Software Association (2013). The 2013 Essential Facts about the Computer and Video Game Industry. https://issuu.com/exame/docs/esa_____essential_facts_about_the_c.

[31] Rea M.S., Figueiro M.G., Bierman A., Bullough J.D. (2010). Circadian light. J. Circadian Rhythm..

[32] Wood B., Rea M.S., Plitnick B., Figueiro M.G. (2013). Light level and duration of exposure determine the impact of self-luminous tablets on melatonin suppression. Appl. Ergon..

[33] Arora T., Broglia E., Thomas G.N., Taheri S. (2014). Associations between specific technologies and adolescent sleep quantity, sleep quality, and parasomnias. Sleep Med..

[34] Morin C.M., Belleville G., Bélanger L., Ivers H. (2011). The Insomnia severity index: Psychometric indicators to detect insomnia cases and evaluate treatment response. Sleep.

[35] National Sleep Foundation (2015). National Sleep Foundation Recommends New Sleep Times. https://www.sleepfoundation.org/press-release/national-sleep-foundation-recommends-new-sleep-times.

[36] Hale L., Guan S. (2015). Screen time and sleep among school-aged children and adolescents: A systematic literature review. Sleep Med. Rev..

[37] Chen Y.L., Gau S.S.F. (2016). Sleep problems and internet addiction among children and adolescents: A longitudinal study. J. Sleep Res..

[38] Gooley J.J., Chamberlain K., Smith K.A., Khalsa S.B.S., Rajaratnam S.M., Van Reen E., Zeitzer J.M., Czeisler C.A., Lockley S.W. (2011). Exposure to room light before bedtime suppresses melatonin onset and shortens melatonin duration in humans. J. Clin. Endocrinol. Metab..

[39] Higuchi S., Motohashi Y., Liu Y., Maeda A. (2005). Effects of playing a computer game using a bright display on presleep physiological variables, sleep latency, slow wave sleep and REM sleep. J. Sleep Res..

[40] Anderson C.A., Bushman B.J. (2001). Effects of violent video games on aggressive behavior, aggressive cognition, aggressive affect, physiological arousal, and prosocial behavior: A meta-analytic review of the scientific literature. Psychol. Sci..

[41] Dworak M., Schierl T., Bruns T., Strüder H.K. (2007). Impact of singular excessive computer game and television exposure on sleep patterns and memory performance of school-aged children. Pediatrics.

[42] Casey B., Jones R.M., Somerville L.H. (2011). Braking and accelerating of the adolescent brain. J. Res. Adolesc..

[43] Steinberg L. (2008). A social neuroscience perspective on adolescent risk-taking. Dev. Rev..

[44] Shulman E.P., Harden K.P., Chein J.M., Steinberg L. (2015). Sex differences in the developmental trajectories of impulse control and sensation-seeking from early adolescence to early adulthood. J. Youth Adolesc..

[45] Weinstein A., Livny A., Weizman A. (2017). New developments in brain research of internet and gaming disorder. Neurosci. Biobehav. Rev..

[46] Acheson A., Richards J.B., de Wit H. (2007). Effects of sleep deprivation on impulsive behaviors in men and women. Physiol. Behav..

[47] Ohayon M.M., Roberts R.E., Zulley J., Smirne S., Priest R.G. (2000). Prevalence and patterns of problematic sleep among older adolescents. J. Am. Acad. Child. Adolesc. Psychiatry.

[48] Suh S., Cho N., Zhang J. (2018). Sex differences in insomnia: From epidemiology and etiology to intervention. Curr. Psychiatry Rep..

[49] Fang J., Fishbein W. (1996). Sex differences in paradoxical sleep: Influences of estrus cycle and ovariectomy. Brain Res..

[50] Müller K.W., Janikian M., Dreier M., Wölfling K., Beutel M., Tzavara C., Richardson C., Tsitsika A. (2015). Regular gaming behavior and internet gaming disorder in European adolescents: Results from a cross-national representative survey of prevalence, predictors, and psychopathological correlates. Eur. Child Adolesc. Psychiatry.

[51] Lemola S., Perkinson-Gloor N., Brand S., Dewald-Kaufmann J.F., Grob A. (2015). Adolescents’ electronic media use at night, sleep disturbance, and depressive symptoms in the smartphone age. J. Youth Adolesc..

[52] Brunborg G.S., Mentzoni R.A., Molde H., Myrseth H., Skouverøe K.J.M., Bjorvatn B., Pallesen S. (2011). The relationship between media use in the bedroom, sleep habits and symptoms of insomnia. J. Sleep Res..

[53] Kim S.-E., Kim J.-W., Jee Y.-S. (2015). Relationship between smartphone addiction and physical activity in Chinese international students in Korea. J. Behav. Addict..

[54] Young K. (2009). Understanding online gaming addiction and treatment issues for adolescents. Am. J. Fam. Ther..

[55] Oulasvirta A., Rattenbury T., Ma L., Raita E. (2012). Habits make smartphone use more pervasive. Pers. Ubiquitous Comput..

[56] Chiu S.-I., Hong F.-Y., Chiu S.-L. (2013). An analysis on the correlation and gender difference between college students’ Internet addiction and mobile phone addiction in Taiwan. Int. Sch. Res. Not..

[57] Ybarra M.L., Mitchell K.J. (2005). Exposure to Internet pornography among children and adolescents: A national survey. Cyberpsychol. Behav..

[58] Lee C., Lee S.-J. (2017). Prevalence and predictors of smartphone addiction proneness among Korean adolescents. Child. Youth Serv. Rev..

[59] Choi S.-W., Kim D.-J., Choi J.-S., Ahn H., Choi E.-J., Song W.-Y., Kim S., Youn H. (2015). Comparison of risk and protective factors associated with smartphone addiction and Internet addiction. J. Behav. Addict..

[60] Ko C.H., Wang P.W., Liu T.L., Yen C.F., Chen C.S., Yen J.Y. (2015). Bidirectional associations between family factors and Internet addiction among adolescents in a prospective investigation. Psychiatry Clin. Neurosci..

